# Research Progress on Chemical Constituents of* Lonicerae japonicae flos*

**DOI:** 10.1155/2016/8968940

**Published:** 2016-06-14

**Authors:** Lingna Wang, Qiu Jiang, Jinghong Hu, Yongqing Zhang, Jia Li

**Affiliations:** ^1^School of Pharmacy, Shandong University of Traditional Chinese Medicine, Jinan 250355, China; ^2^Shiyan Centers for Disease Control and Prevention, Shiyan 442012, China

## Abstract

*Lonicerae japonicae flos* is commonly used in traditional Chinese medicine for thousands of years with confirmed curative effects. Except for medicine, it is also used in healthy food, cosmetics, and soft beverages for its specific activities. Therefore, the chemical constituents, mainly including organic acids, flavonoids, iridoids, triterpenoids, and volatile oils, have been well studied by many scholars in recent years and a comprehensive and systematic review on chemical constituents of* Lonicerae japonicae flos* is indispensable. This paper aims at reviewing the chemical components of LJF in recent years through searching for the literatures both at home and abroad. Our results show that 212 components have been isolated from* Lonicerae japonicae flos*, including 27 flavonoids, 40 organic acids, 83 iridoids, 17 triterpenoids, and 45 other compounds, which could lay a foundation for the further application of* Lonicerae japonicae flos*.

## 1. Introduction

*Lonicerae japonicae flos* (LJF, also Jinyinhua in Chinese), coming from the dried buds of* Lonicera japonica* Thunb., has been widely used in traditional Chinese medicine (TCM) for several thousands of years due to its heat-clearing and detoxifying properties. In clinical practice, more than 500 prescriptions including LJF have been used to treat various diseases [1]. Pharmacological studies show that LJF possessed various actions, such as anti-inflammatory, antiviral, antidiabetic, antiallergic, and antioxidants [2–5], and could be used to treat many viral diseases, such as SARS and H7N9 virus and infections [6–9]. In addition, it is also used as healthy food, cosmetics, soft beverages, and ornamental groundcover, for its specific activities [10–13]. Therefore, many scholars inside and outside have drawn great attention on LJF in recent years and they have isolated a lot of chemical components from LJF, such as organic acids, flavones, iridoids, triterpenoids, and volatile oils [14], which have been reported as the active constituents with some potential pharmacological effects. Therefore, a comprehensive and systematic review on chemical constituents of LJF is indispensable.

Taking the abovementioned consideration, this paper comprehensively reviews chemical constituents of the dried flower buds of* Lonicera japonica* Thunb., in order to lay a foundation for the further study of LJF.

## 2. Constituents

More than 212 compounds have been isolated and identified from LJF so far, including organic acids, flavonoids, iridoids, triterpenoids, and volatile oils, which are the material basis of* Lonicerae japonicae flos's* pharmacological effects and constitute the primary effective substances. Beyond that, other groups of compounds were also reported.

### 2.1. Flavonoids

**Figure 1:**
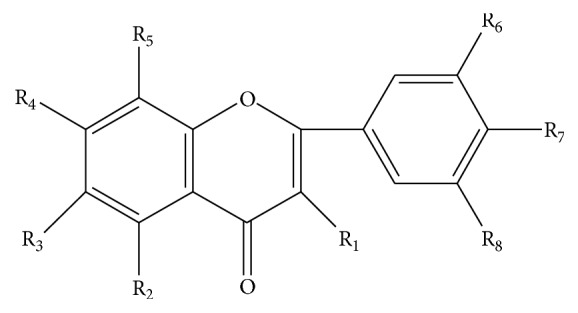
Skeleton of flavonoids.

Up to now, 27 flavonoids have been isolated from LJF, mainly including quercetin (**1**), rutin (**2**), luteolin-7-O-*β*-D-glucopyranoside (**3**), kaempferol-3-O-*β*-D-glucopyranoside (**4**), apigenin-7-O-*α*-L-rhamnopyranoside (**5**), chrysoeriol-7-O-*β*-D-glucopyranosyl (**6**), luteolin-3′-L-rhamnoside (**7**), luteolin (**8**), flavoyadorinin-B (**9**), rhoifolin (**10**), quercetin-3-O-*β*-D-glucopyranoside (**11**), 3′-methoxy luteolin (**12**), 5,3′-dimethoxy luteolin (**13**), luteolin-5-O-*β*-D-glucopyranoside (**14**), apigenin (**15**), isorhamnetin-3-O-*β*-D-glucopyranoside (**16**), hyperoside (**17**), quercetin-7-O-*β*-D-glucopyranoside (**18**), kaempferol-3-O-*β*-D-rutinoside (**19**), isorhamnetin-3-O-*β*-D-rutinoside (**20**), 5-hydroxyl-3′,4′,7-trimethoxy flavone (**21**), 5-hydroxyl-6,7,8,4′-tetramethoxy flavone (**22**), corymbosin (**23**), 5-hydroxyl-7,4′-dimethoxy flavone (**24**), lonicerin (**25**), 5,7,3′,4′,5′-pentamethoxy flavone (**26**), and 5,4′-dihydroxy-3′,5′-dimethoxy-7-*β*-D-glucoxy-flavone (**27**). The structures of 27 flavonoids have been given in Figure 1 and Table 1.

### 2.2. Organic Acids

**Figure 2:**
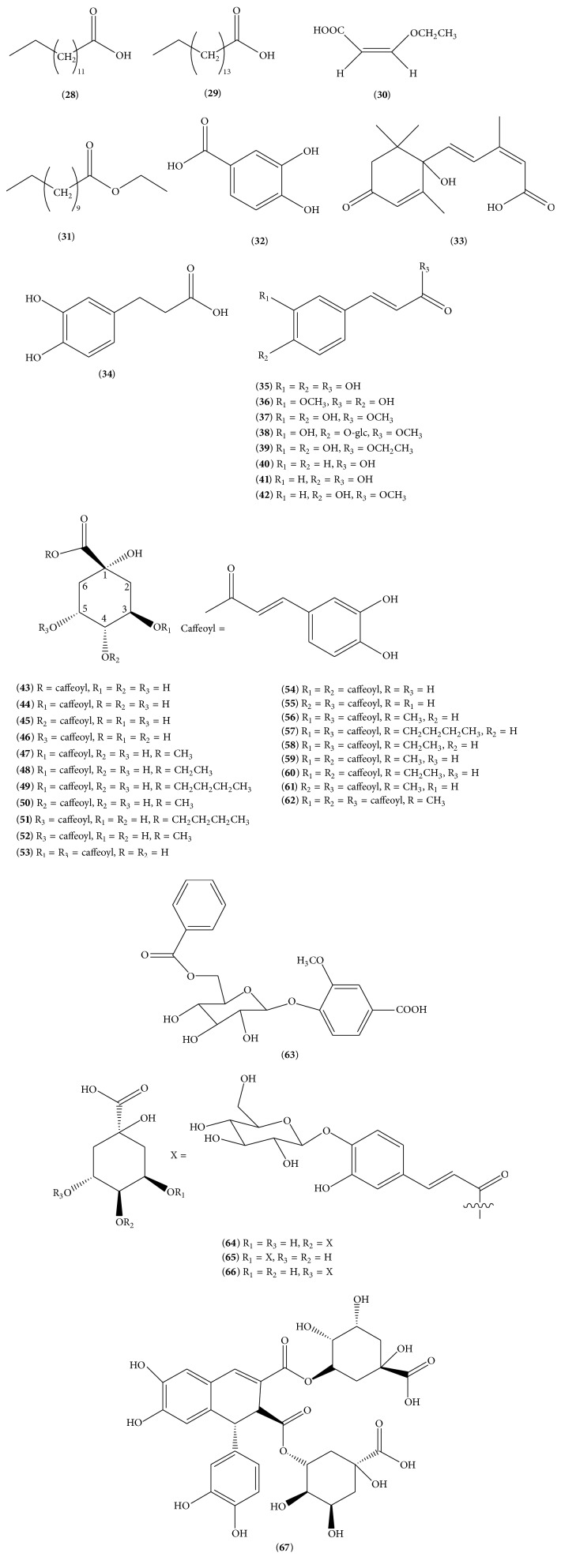
The structures of 40 organic acids (**28**)–(**67**).

Just like many other herbs, LJF is also a rich source of organic acids, and until now, more than 40 organic acids (**28**–**67**) have been isolated from LJF, the structures of which are shown in Figure 2. The organic acids mainly include myristic acid (**28**), palmitic acid (**29**) [15], 2(E)-3-ethoxy acrylic acid (**30**) [16, 17], ethyl laurate (**31**), protocatechuic acid (**32**) [18], abscisic acid (**33**) [19], 3-(3, 4-dihydroxyphenyl) propionic acid (**34**) [20], caffeic acid (**35**), ferulic acid (**36**) [11], caffeic acid methyl ester (**37**) [20], methyl 4-O-*β*-D-glucopyranosyl caffeate (**38**) [21], caffeic acid ethyl ester (**39**) [18], cinnamic acid (**40**) [22], 4-hydroxycinnamic acid (**41**), and methyl 4-hydroxycinnamate (**42**) [20]. About 20 caffeic acid derivatives are isolated from LJF extract, including 1-O-caffeoylquinic acid (**43**) [23], 3-O-caffeoylquinic acid (**44**) [24], 4-O-caffeoylquinic acid (**45**), 5-O-caffeoylquinic acid (**46**) [25], 3-O-caffeoylquinic acid methyl ester (**47**), 3-O-caffeoylquinic acid ethyl ester (**48**) [26, 27], 3-O-caffeoylquinic acid butyl ester (**49**) [28], 4-O-caffeoylquinic acid methyl ester (**50**) [21], 5-O-caffeoylquinic acid butyl ester (**51**), 5-O-caffeoylquinic acid methyl ester (**52**), 3,5-O-dicaffeoylquinic acid (**53**), 3,4-O-dicaffeoylquinic acid (**54**), 4,5-O-dicaffeoylquinic acid (**55**), 3,5-O-dicaffeoylquinic acid methyl ester (**56**) [26, 27], 3,5-O-dicaffeoylquinic acid butyl ester (**57**) [29], 3,5-O-dicaffeoylquinic acid ethyl ester (**58**), 3,4-O-dicaffeoylquinic acid methyl ester (**59**), 3,4-O-dicaffeoylquinic acid ethyl ester (**60**), 4,5-O-dicaffeoylquinic acid methyl ester (**61**) [30], and 3,4,5-O-tricaffeoylquinic acid (**62**) [27]. In addition, some special organic acids, such as vanillic acid, 4-O-*β*-D-(6-O-benzoylglucopyranoside) (**63**) [26], (−)-4-O-(4-O-*β*-D-glucopyranosylcaffeoyl) quinic acid (**64**), (−)-3-O-(4-O-*β*-D-glucopyranosylcaffeoyl) quinic acid (**65**), (−)-5-O-(4-O-*β*-D-glucopyranosylcaffeoyl) quinic acid (**66**) [31], and dichlorogelignate (**67**) [21], have been obtained.

### 2.3. Iridoids

**Figure 3:**
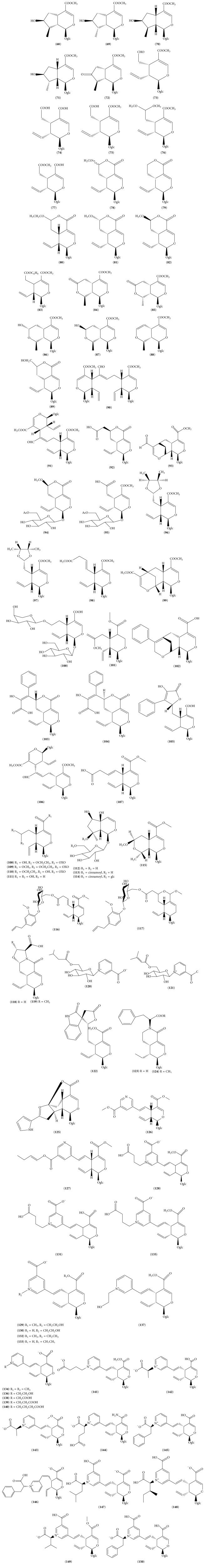
The structures of 83 iridoids (**68**)–(**150**).

Iridoids are the most abundant compounds in LJF. Thus far, more than 83 iridoids have been isolated from LJF, which can be classified into three classes: iridoid glucosides, secoiridoid glycosides, and N-contained iridoid glycosides. Among them, six iridoid glucosides, such as loganin (**68**), 8-epiloganin (**69**), loganic acid (**70**), 8-epiloganic acid (**71**), and ketologanin (**72**) [32], have been isolated from LJF. Meanwhile, 47 secoiridoid glycosides (**73**–**117**) also are identified from LJF, including secologanin (**73**), secologanoside (**74**), secoxyloganin (**75**) [19], secologanin dimethyl acetal (**76**) [32], secologanoside-7-methyl ester (**77**) [33], secologanic acid (**78**), sweroside (**79**), 7-O-ethylsweroside (**80**), vogeloside (**81**), 7-epi-vogeloside (**82**) [25], secoxyloganin-7-butyl ester (**83**) [34], kingiside (**84**) [21], 8-epikingiside (**85**) [32], 7*α*-morroniside (**86**), 7*β*-morroniside (**87**) [21], dehydromorroniside (**88**) [35], 7-hydroxy-methyl-vogeloside (**89**) [17], (Z)-aldosecologanin (**90**), (E)-aldosecologanin (**91**) [32], loniaceticiridoside (**92**), lonimalondialiridoside (**93**) [36], 6′-O-acetylvogeloside (**94**), 6′-O-acetylsecoxyloganin (**95**) [22], loniceracetalide A (**96**), loniceracetalide B (**97**) [33], adinoside A (**98**), stryspinoside (**99**) [19], secologanoside A (**100**) [37], dimethyl secologanoside (**101**) [38], loniphenyruviridoside A~D (**102**–**105**) [39], centauroside (**106**) [23], loniceranan A (**107**), loniceranan B (**108**), loniceranan C (**109**), ethyl secologanoside (**110**), demethylsecologanol (**111**), harpagide (**112**), harpagoside (**113**), 6′′-O-*β*-glucopyranosylharpagoside (**114**), (7*β*)-7-O-methyl morroniside (**115**) [32], lonicerjaponin A (**116**), and lonicerjaponin B (**117**) [40]. 33 N-contained iridoid glycosides (**118**–**150**) have been isolated from LJF in recent years, including serinosecologanin (**118**), threoninosecologanin (**119**) [41], lonijaponinicotinosides A (**120**), lonijaponinicotinosides B (**121**) [42], lonijapospiroside A (**122**), L-phenylalaninosecologanin B (**123**), L-phenylalaninosecologanin C (**124**), and dehydroprolinoylloganin A (**125**) [30]. In 2013, Kashiwada et al. isolated two conjugates with a nicotinic acid derivative (**126**-**127**) [40]. Additionally, in 2008, Song et al. isolated three pyridinium alkaloid-coupled secoiridoids from an aqueous extract of the flower buds of* Lonicera japonica*, lonijaposides A–C (**128**–**130**) [43]. In 2011, Yu et al. isolated lonijaposides D–N (**131**–**141**) [39] and, in 2013, Yu et al. obtained lonijaposides O–W (**142**–**150**) from an aqueous extract of the flower buds of* Lonicera japonica* Thunb. [44]. The structures of 83 iridoids are listed in Figure 3.

### 2.4. Triterpenoids

**Figure 4:**
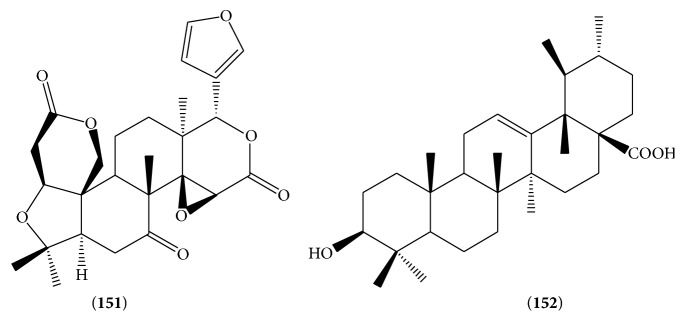
The structures of compounds of (**151**)-(**152**).

**Figure 5:**
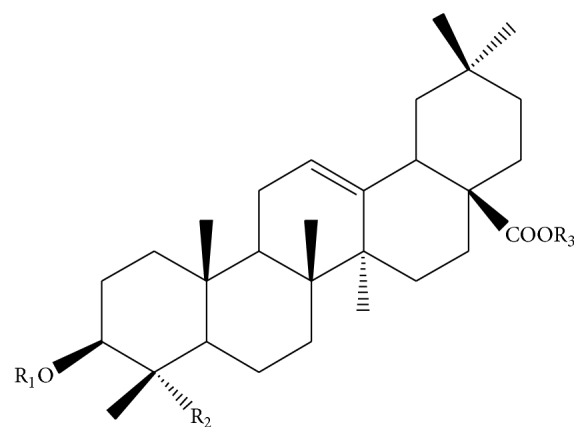
Skeleton of triterpenoids.

17 triterpenoids are found from LJF and their structures are listed in Figures 4 and 5 and Table 2, mainly including limonin (**151**) [45], ursolic acid (**152**) [22], and oleanolic acid triterpenoid saponins (**153**–**156**) and hederagenin triterpenoid saponins (**157**–**167**). Oleanolic acid triterpenoid saponins include oleanolic acid (**153**), 3-O-*β*-D-glucopyranosyl-(1→2)-*α*-L-arabinopyranosyl oleanolic acid-28-O-*β*-D-glucopyranosyl-(1→6)-*β*-D-glucopyranoside (**154**), oleanolic acid 28-O-*α*-L-rhamnopyranosyl-(1→2)-[*β*-D-xylopyranosyl(1→6)]-*β*-D-glucopyranosyl ester (**155**), loniceroside E (**156**), hederagenin 3-O-*α*-L-arabinopyranoside (**157**), loniceroside D (**158**), loniceroside A (**159**), loniceroside B (**160**), loniceroside C (**161**), 3-O-*β*-D-glucopyranosyl(1→4)-*β*-D-glucopyranosyl(1→3)-*α*-L-rhamnopyranosyl(1→2)-*α*-L-arabinopyranosyl-hederagenin-28-O-*β*-D-glucopyranosyl(1→6)-*β*-D-glucopyranosyl ester (**162**), hederagenin-3-O-*α*-L-rhamnopyranosyl(1→2)-*α*-L-arabinopyranoside (**163**), 3-O-*α*-L-rhamnopyranosyl(1→2)-*α*-L-arabinopyranosyl-hederagenin-28-O-*β*-D-xylopyranosyl(1→6)-*β*-D-glucopyranosyl ester (**164**), 3-O-*α*-L-rhamnopyranosyl(1→2)-*α*-L-arabinopyranosyl-hederagenin-28-O-*β*-D-glucopyranosyl(1→6)-*β*-D-glucopyranosyl ester (**165**), 3-O-*α*-L-rhamnopyranosyl(1→2)-*α*-L-arabinopyranosyl-hederagenin-28-O-*β*-D-rhamnopyranosyl(1→2)-[*β*-D-xylopyranosyl(1→6)]-*β*-D-glucopyranosyl ester (**166**), and 3-O-*β*-D-glucopyranosyl(1→3)-*α*-L-rhamnopyranosyl(1→2)-*α*-L-arabinopyranosyl-hederagenin-28-O-*β*-D-glucopyranosyl(1→6)-*β*-D-glucopyranosyl ester (**167**).

### 2.5. Volatile Oils

Volatile oils, one of the important effective constituents of LJF, play a significant role on the pharmacological effects, which are also used in cosmetics, spices, and other industries. There are some differences of volatile oils components between different groups and different germplasms, mainly including alkylation, alcohol, alkene, and ketone. Du et al. [46] identified 35 volatile constituents in LJF from Guangxi Zhuang Autonomous Region, mainly including methyl linolenate, n-hexadecanoic acid, and *ε*-muurolene, and 18 volatile constituents in LJF from Hunan province, mainly including n-hexadecanoic acid, linoleic acid, and *α*-curcumene. Yang and Zhao [47] identified 49 volatile constituents in LJF from Ningxia province; three major components are linalool (13.59%), carvacrol (7.67%), and dibutyl phthalate (7.54%). Guan et al. [48] investigated the chemical constituents of essential oil in the fresh and dried buds of LJF “Jiu Feng 1,” and 44 volatile constituents were identified from the fresh buds, mainly including lower boiling point chemical compounds, such as linalool (5.21%), farnesol (2.60%), ascorbyl dipalmitate (9.49%), and nonacosane (17.38%), and 49 volatile constituents from the dried buds, mainly including higher boiling point chemical compounds, such as methyl hexadecanoate (13.99%) and methyl linolenate (9.20%). This may be chemical constituent changes from fresh buds and dried buds caused by different natural drying process. In addition, methods of extraction can also affect the class and content of the volatile oils. Du et al. [49] extracted volatile oils from LJF using steam distillation and fresh flowers homogenate extraction, respectively, and then extracted by diethyl ether and analyzed constituents by GC-MS. Results show that volatile oils extracted by fresh flowers homogenate extraction mainly include benzenepropanal (12.4%), ethylbenzene (8.58%), benzaldehyde (8.04%), linalool oxide trans (4.72%), and isophytol (2.94%), and volatile oils extracted by steam distillation mainly include cyclohexanol (8.06%), oxalic acid, cyclohexyl isobutyl ester (3.45%), cyclohexane-cyclopentylmethyl (18.35%), n-hexadecanoic acid (12.56%), and benzene cyclohexylmethyl (9.77%). The result shows that there are great differences between the compositions of volatile oils before and after heat treatment, which provides a new way of thinking for the use of fresh buds of LJF.

### 2.6. Others

**Figure 6:**
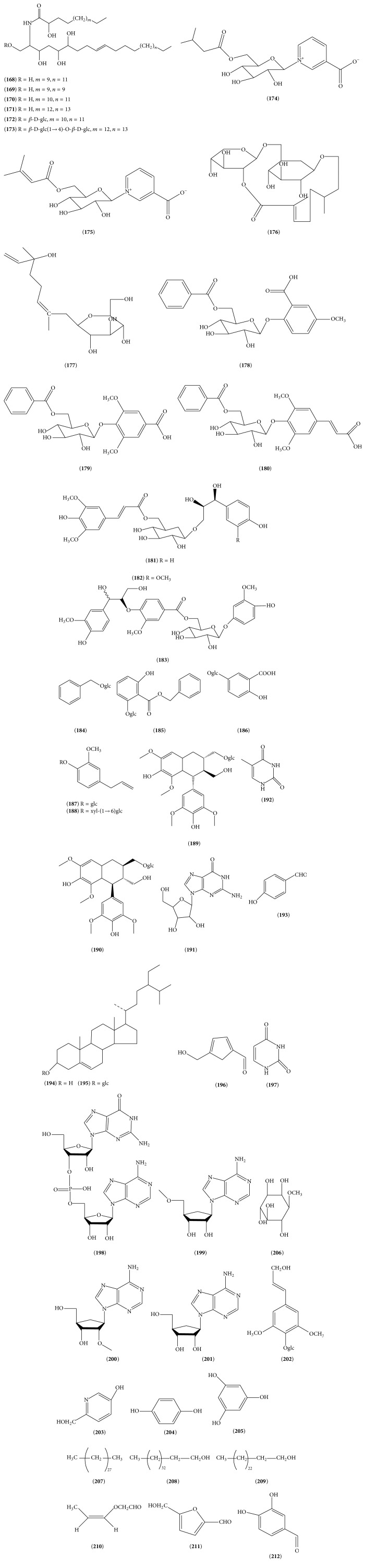
The structures of compounds of (**168**)–(**212**).

Other chemical constituents other than organic acids, flavonoids, iridoids, triterpenoids, and volatile oils were also found in LJF. In 2006, Kumar et al. [50] isolated six novel cerebrosides, lonijaposides A_1_–A_4_, B_1_, and B_2_ (**168**–**173**) from the flowers of* Lonicera japonica* Thunb. In 2008, Song [51] identified two nicotinic acids N-glycosides, (+)-N-(3-methybutyryl-*β*-D-glucopyranoyl)-nicotinate (**174**) and (+)-N-(3-methybut-2-enoyl-*β*-D-glucopyranoyl)-nicotinate (**175**). In 2008, Wang [52] isolated (2E)-(6S)-8-[*α*-L-arabinopyranosyl-(1′′ → 6′)-*β*-D-glucopyranosyloxy]-2,6-dimethyloct-2-eno-1,2′′-lactone (**176**) and 2,6-dimethyl-6-hydroxyl-2,7-diene-1-octyl alcoholglucopyranoside (**177**) from the flowers of* Lonicera japonica*. In 2013, Wang et al. [53] isolated six new glycosides from the flower buds of* Lonicera japonica* Thunb. These are (−)-2-hydroxy-5-methoxybenzoic acid 2-O-*β*-D-(6-O-benzoyl)-glucopyranoside (**178**), (−)-4-hydroxy-3,5-dimethoxybenzoic acid 4-O-*β*-D-(6-O-benzoyl)-glucopyranoside (**179**), (−)-(E)-3,5-dimethoxyphenylpropenoic acid 4-O-*β*-D-(6-O-benzoyl)-glucopyranoside (**180**), (−)-(7S,8R)-4-hydroxyphenylglycerol 9-O-*β*-D-[6-O-(E)-4-hydroxy-3,5-dimethoxyphenylpropenoyl]-glucopyranoside** (181)**, (−)-(7S,8R)-4-hydroxy-3-methoxyphenylglycerol 9-O-*β*-D-[6-O-(E)-4-hydroxy-3,5-dimethoxyphenylpropenoyl]-glucopyranoside** (182)**, and (−)-4-hydroxy-3-methoxyphenol *β*-D-{6-O-[4-O-(7S,8R)-(4-hydroxy-3-methoxyphenylglycerol-8-yl)-3-methoxybenzoyl]}-glucopyranoside (**183**). At the same year, Wang et al. [19] isolated benzyl alcohol *β*-D-glucoside (**184**), benzyl 2-O-*β*-D-glucopyranosyl-2,6-dihydroxybenzoate (**185**), gentisic acid 5-O-*β*-D-glucopyranoside (**186**), eugenyl *β*-D-glucopyranoside (**187**), eugenyl *β*-D-xylopyranosyl-(1→6)-*β*-D-glucopyranoside (**188**), (−)-lyoniresinol 9-O-*β*-D-glucopyranoside (**189**), (+)-lyoniresinol 9-O-*β*-D-glucopyranoside (**190**), guanosine (**191**), 5-methyluracil (**192**), p-hydroxybenzaldehyde (**193**), *β*-sitosterol (**194**), daucosterol (**195**), 5-hydroxymethyl-2-furancarboxaldehyde (**196**), and uracil (**197**) from LJF. In 2015, Yu et al. [21] identified guanosinyl-(3′ → 5′)-adenosine monophosphate (**198**), 5′-O-methyladenosine (**199**), 2′-O-methyladenosine (**200**), adenosine (**201**), syringing (**202**), and 6-hydroxymethyl-3-pyridinol (**203**). Additionally, P-hydroxy-phenol (**204**), 1,2,4-benzenetriol (**205**) [20], 1-O-methyl-myo-inositol (**206**), nonacontane (**207**) [28], pentatriaconta alcohol (**208**), pentacosa alcohol (**209**), 2-(2-propenyloxy)-ethanal (**210**) [54], 5-hydroxymethyl-2-furfural (**211**) [55], and 3,4-dihydroxybenzaldehyde (**212**) [15] were also isolated. The structures of compounds of (**168**)–(**212**) are shown in Figure 6.

Certainly, some other constituents, such as proteins and amino acids, have been obtained from LJF, which were also found to be rich in metal elements, such as Ca, Mg, Mn, Cu, Fe, and Zn [56, 57].

## 3. Discussion and Conclusion

LJF is a widely used medicine which has been demonstrated to be useful for the treatment and prevention of severe acute respiratory syndromes, H1N1 influenza and hand-foot-and-mouth disease. The present review summarizes the chemical constituents of LJF found in recent years, and the results indicate that more than 212 components have been identified from extracts of LJF, which contain 27 flavonoids, 40 organic acids, 83 iridoids, 17 triterpenoids, and 45 other compounds. We can clearly see that LJF has complex chemical composition resulting in good clinical efficacy due to the interactions among the components. However, only chlorogenic acid and luteoloside are used as biomarkers in Chinese Pharmacopoeia in 2015 edition for evaluating the quality of LJF. At a certain stage, it cannot comprehensively inflect the quality of LJF and further studies of chemical constituents and pharmacological effects of LJF ought to be conducted, which could lay a foundation for the further application of LJF.

## Figures and Tables

**Table 1:** The structures of compounds (1)–(27) isolated from LJF.

Comp. number	Substitutional groups								References
	1	2	3	4	5	6	7	8	
(1)	OH	OH	H	OH	H	OH	OH	H	[25]
(2)	O-glc-rha	OH	H	OH	H	OH	OH	H	[25]
(3)	H	OH	H	O-glc	H	OH	OH	H	[25]
(4)	O-glc	OH	H	OH	H	H	OH	H	[19, 25]
(5)	H	OH	H	O-rha	H	H	OH	H	[19]
(6)	H	OH	H	O-glc	H	OCH_3_	OH	H	[19]
(7)	H	OH	H	OH	H	O-rha	OH	H	[19]
(8)	H	OH	H	OH	H	OH	OH	H	[58]
(9)	H	H	H	OCH_3_	H	O-glc	OCH_3_	H	[26]
(10)	H	OH	H	O-rha-glc	H	H	OH	H	[26]
(11)	O-glc	OH	H	OH	H	OH	OH	H	[26]
(12)	H	OH	H	OH	H	OCH_3_	OH	H	[20]
(13)	H	OCH_3_	H	OH	H	OCH_3_	OH	H	[20]
(14)	H	O-glc	H	OH	H	OH	OH	H	[20]
(15)	H	OH	H	OH	H	H	OH	H	[20]
(16)	O-glc	OH	H	OH	H	OCH_3_	OH	H	[59]
(17)	O-gal	OH	H	OH	H	OH	OH	H	[59]
(18)	OH	OH	H	O-glc	H	OH	OH	H	[60]
(19)	O-glc-rha	OH	H	OH	H	H	OH	H	[28]
(20)	O-glc-rha	OH	H	OH	H	OCH_3_	OH	H	[28]
(21)	H	OH	H	OCH_3_	H	OCH_3_	OCH_3_	H	[15]
(22)	H	OH	OCH_3_	OCH_3_	OCH_3_	H	OCH_3_	H	[18]
(23)	H	OH	H	OCH_3_	H	OCH_3_	OCH_3_	OCH_3_	[61]
(24)	H	OH	H	OCH_3_	H	H	OCH_3_	H	[61]
(25)	H	OH	H	O-rha-glc	H	OH	OH	H	[61]
(26)	H	OCH_3_	H	OCH_3_	H	OCH_3_	OCH_3_	OCH_3_	[62]
(27)	H	OH	H	O-glc	H	OCH_3_	OH	OCH_3_	[45]

**Table 2:** The structures of compounds (153)–(167) obtained from LJF.

Comp. number	Substituent groups	References
(**153**)	R_1_ = R_3_ = H, R_2_ = CH_3_	[28]
(**154**)	R_1_ = glc-(1→2)-ara, R_2_ = CH_3_ R_3_ = glc-(1→6)-glc	[61]
(**155**)	R_1_ = H, R_2_ = CH_3_ R_3_ = rha-(1→2)-[xyl-(1→6)]-glc	[55]
(**156**)	R_1_ = glc, R_2_ = CH_3_, R_3_ = rha(1→2)[xyl(1→6)]glc	[24, 63]
(**157**)	R_1_ = ara, R_2_ = CH_2_OH, R_3_ = H	[55]
(**158**)	R_1_ = glc, R_2_ = CH_2_OH, R_3_ = glc(1→2)[xyl(1→6)]glc	[24, 63]
(**159**)	R_1_ = ara, R_2_ = CH_2_OH, R_3_ = rha(1→2)[xyl(1→6)]glc	[24, 63]
(**160**)	R_1_ = rha, R_2_ = CH_2_OH, R_3_ = rha(1→2)[xyl(1→6)]glc	[24]
(**161**)	R_1_ = glc, R_2_ = CH_2_OH, R_3_ = rha(1→2)[xyl(1→6)]glc	[24, 63]
(**162**)	R_1_ = glc(1→4)glc(1→3)rha(1→2)ara, R_2_ = CH_2_OH, R_3_ = glc(1→6)glc	[64]
(**163**)	R_1_ = rha(1→2)ara, R_2_ = CH_2_OH, R_3_ = H	[64]
(**164**)	R_1_ = rha(1→2)ara, R_2_ = CH_2_OH, R_3_ = xyl(1→6)glc	[64]
(**165**)	R_1_ = rha(1→2)ara, R_2_ = CH_2_OH, R_3_ = glc(1→6)glc	[64]
(**166**)	R_1_ = rha(1→2)ara, R_2_ = CH_2_OH, R_3_ = rha(1→2)xyl(1→6)glc	[64]
(**167**)	R_1_ = glc(1→3)rha(1→2)ara, R_2_ = CH_2_OH, R_3_ = glc(1→6)glc	[64]
